# Molecular characterization and immune protection of the 3-hydroxyacyl-CoA dehydrogenase gene in *Echinococcus granulosus*

**DOI:** 10.1186/s13071-021-05001-z

**Published:** 2021-09-23

**Authors:** Jinwen Xian, Ning Wang, Pengpeng Zhao, Yanyan Zhang, Jimeng Meng, Xun Ma, Xiaola Guo, Zhengrong Wang, Xinwen Bo

**Affiliations:** 1 State Key Laboratory of Sheep Genetic Improvement and Healthy Production/Institute of Animal Husbandry and Veterinary Medicine, Xinjiang Academy of Agricultural and Reclamation Sciences, 832000 Shihezi, China; 2 College of Animal Science and Technology, Shihezi University, Shihezi, 832000 China; 3 State Key Laboratory of Veterinary Etiological Biology, Key Laboratory of Veterinary Parasitology of Gansu Province, Lanzhou Veterinary Research Institute, Chinese Academy of Agricultural Sciences, Gansu, 730046 China

**Keywords:** *E. granulosus*, Cystic echinococcosis, *EgHCDH*, Immunolocalization, Immunogenicity, Dog vaccine

## Abstract

**Background:**

Cystic echinococcosis (CE) is a serious parasitic zoonosis caused by the larvae of the tapeworm *Echinococcus granulosus*. The development of an effective vaccine is one of the most promising strategies for controlling CE.

**Methods:**

The *E. granulosus* 3-hydroxyacyl-CoA dehydrogenase (*EgHCDH*) gene was cloned and expressed in *Escherichia coli.* The distribution of EgHCDH in protoscoleces (PSCs) and adult worms was analyzed using immunofluorescence. The transcript levels of *EgHCDH* in PSCs and adult worms were analyzed using quantitative real-time reverse transcription PCR (RT-qPCR). The immune protective effects of the rEgHCDH were evaluated.

**Results:**

The 924-bp open reading frame sequence of *EgHCDH*, which encodes a protein of approximately 34 kDa, was obtained. RT-qPCR analysis revealed that *EgHCDH* was expressed in both the PSCs and adult worms of *E. granulosus*. Immunofluorescence analysis showed that EgHCDH was mainly localized in the tegument of PSCs and adult worms. Western blot analysis showed that the recombinant protein was recognized by *E. granulosus*-infected dog sera. Animal challenge experiments demonstrated that dogs immunized with recombinant (r)EgHCDH had significantly higher serum IgG, interferon gamma and interleukin-4 concentrations than the phosphate-buffered saline (PBS) control group. The rEgHCDH vaccine was able to significantly reduce the number of *E. granulosus* and inhibit the segmental development of *E. granulosus* compared to the PBS control group.

**Conclusions:**

The results suggest that rEgHCDH can induce partial immune protection against infection with *E. granulosus* and could be an effective candidate for the development of new vaccines.

**Graphical abstract:**

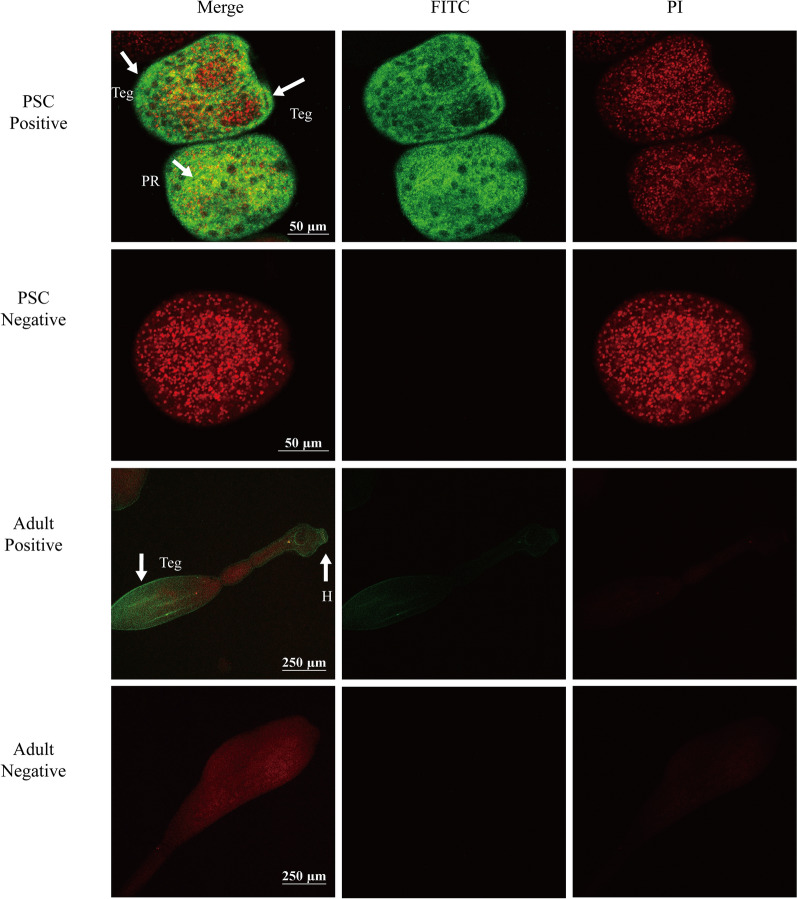

**Supplementary Information:**

The online version contains supplementary material available at 10.1186/s13071-021-05001-z.

## Background

Cystic echinococcosis (CE) is a zoonotic parasitic disease caused by larvae of the tapeworm *Echinococcus granulosus* [1]. Dogs are the main definitive hosts of *E. granulosus*. In recent years, the number of stray dogs has increased annually, which has greatly increased the risk of transmission of CE [2]. Mammals, such as sheep, cattle, camels and donkeys, are critical intermediate hosts in China [3]. Humans are infected by accidentally ingesting food or water containing *E. granulosus* eggs [4]. CE is a serious threat to human health and is a burden to patients and the economy [5, 6]. The World Health Organization has listed CE as one of the 17 neglected diseases in the 2020 strategic roadmap because of its prevalence and severity [7]. CE control currently relies primarily on prevention and the use of anticoccidial drugs and vaccines. Drugs, such as praziquantel, are used mainly to deworm the definitive hosts (dogs) in order to block the transmission of *E. granulosus.* However, deworming with these drugs is time-consuming and difficult to implement on a large scale in epidemic areas, and there is also the risk of the development of drug resistance. To date, the recombinant Eg95 vaccine is able to protect sheep and other intermediate host species against CE [8]. However, the Eg95 vaccine had no protective effect on infected animals with cysts. Therefore, the development of a dog vaccine against *E. granulosus* is urgently needed.

Cestodes lack a digestive tract, and all tapeworms must transport nutrients from the host through the tegumental syncytium [1]. Analysis of the genomic data of *E. granulosus* revealed that it lacks the ability for de novo synthesis of lipids, purines and most amino acids, but it has a complete set of tricarboxylic acid cycle enzymes (TCA) [9]. Among these enzymes, 3-hydroxyacyl-CoA dehydrogenase (HCDH) is one of the main functional enzymes in the β-oxidation process [9, 10]. In this study, to further explore the role of *E. granulosus* *HCDH* (*EgHCDH*) in the development of *E. granulosus* and to evaluate its potential as a vaccine antigen candidate, we cloned and molecularly characterized the gene encoding HCDH in *E. granulosus* (*EgHCDH*). We also evaluated the immunogenicity of recombinant (r)EgHCDH in a dog challenge model.

## Methods

### Ethics statement

This study was reviewed and approved by the Care and Use of Laboratory Animals Department of the Xinjiang Academy of Agricultural and Reclamation Sciences (Shihezi, China) (Approval No. 2019-012, April 9, 2019). All animals were handled in strict accordance with the animal protection laws of the People’s Republic of China (a draft of the animal protection law was released on September 18, 2009) and the National Standards for Laboratory Animals in China (executed on January 5, 2002).

### Animals

Female BALB/c mice aged 6–8 weeks were purchased from the Laboratory Animal Center of Xin Jiang Medical University (Xingjiang, China). Nine 8-month-old beagle dogs of mixed breed were obtained from the Institute of Musk Deer Breeding.

### Parasites

Hydatid cysts were collected from the livers of naturally infected sheep at an abattoir in Urumqi, Xinjiang Province, China. The fertility of the cysts was confirmed by observing the protoscoleces (PSCs) within the cysts under light microscopy. Once identified, the PSCs were separated and treated as previously described [11]. Briefly, 2000 PSCs were cultured in 1 ml of Roswell Park Memorial Institute 1640 medium with 10% bovine serum albumin (Hyclone, Logan, UT, USA), 100 U/ml penicillin and 100 μg/ml streptomycin (Sigma-Aldrich, St. Louis, MO, USA). *Echinococcus granulosus* adult worms were obtained from an 8-month-old dog 28 days after being artificially infected with PSCs.

### Bioinformatics analysis

The cDNA sequence of *EgHCDH* was downloaded from the National Centre for Biotechnology Information (NCBI), and the physicochemical parameters were analyzed using ProtParam of ExPASY (https://web.expasy.org/protparam/). The open reading frames (ORFs) of *EgHCDH* were analyzed using ORF Finder (https://www.ncbi.nlm.nih.gov/orfnder/). Signal peptides and transmembrane areas were predicted using the SignalP server available online (http://www.cbs.dtu.dk/services/SignalP-3.0/) and the TMHMM-2.0 software package (http://www.cbs.dtu.dk/services/TMHMM-2.0/). Tertiary (three-dimensional) structures were modeled using SWISS-MODEL (http://swissmodel.expasy.org/).

*EgHCDH*-similar sequences were aligned, and phylogenetic trees were constructed using MEGA software (version 5.05) with the neighbor-joining method [12].

### Expression and purification of *EgHCDH*

Total RNA was extracted using an RNA prep Pure Tissue Kit (Nanjing Vazyme Biotech, Nanjing, China). First-strand cDNA was synthesized using a reverse transcription system kit (Nanjing Vazyme Biotech). The full coding sequence of the non-membrane region of *EgHCDH* was amplified using the primers 5′- CGG GAT CCA TGT CAG CCG GTG CTG G-3′ (*Bam*HI) and 5′-GAC GTC GAC TCA CTG TTT TTC CTT GAC AAT GCG C-3′ (*Sal*I). Amplification reactions were performed using the following cycling conditions: pre-denaturation at 95 °C, 5 min; then denaturation at 95 °C/30 s, 62 °C/30 s, 72 °C/1 min; with a final extension at 72 °C, 5 min. Through sequencing, digestion and ligation, *EgHCDH* was ligated into the pET32a (+) plasmid (Novagen, Darmstadt, Germany) and transformed into *Escherichia coli* BL21 (DE3) cells (Tiangen, Beijing, China). Protein expression was induced with 1 mM isopropyl-1-thio-β-d-galactopyranoside at 37 °C for 6 h. The rEgHCDH protein was purified using Ni^2+^ affinity chromatography (Bio-Rad, Hercules, CA, USA), with the the purity of the final product determined by 10% sodium dodecyl sulfate–polyacrylamide gel electrophoresis (SDS-PAGE). The concentrations of the purified protein were determined using a NanoDrop 2000c spectrophotometer (Bio-Rad).

### Preparation of polyclonal antibodies

Each mouse was subcutaneously immunized with 50 µg rEgHCDH emulsified in Freund’s complete adjuvant (Sigma-Aldrich), followed by three repeated inoculations with 50 µg rEgHCDH emulsified in Freund’s incomplete adjuvant (Sigma-Aldrich) every 2 weeks. Control mice were immunized with phosphate-buffered saline (PBS). Sera were collected at post-immunization days 0, 14, 28, 42 and 56.

### Western blotting

Total proteins extracted from PSCs and rEgHCDH protein were separated by 10% SDS-PAGE and then transferred onto PVDF membranes. The membranes were blocked in 5% (w/v) skim milk at 37 °C for 2 h and then incubated with *E. granulosus*-infected dog sera or anti-rEgHCDH mouse sera (1:100 v/v dilutions) overnight at 4 °C. The membranes were then washed and incubated with horseradish peroxidase (HRP)-conjugated sheep anti-mouse IgG or rabbit anti-dog IgG (1:5000 v/v dilution) for 2 h. Signals were visualized using an ECL kit (Pierce ECL Western Blotting Substrate; Thermo Scientific, Waltham, MA, USA) and a molecular imaging system (Bio-Rad).

### Reverse-transcription quantitative PCR

Total RNA was extracted from PSCs and strobilated worms, and cDNA was synthesized as described above. Reverse-transcription quantitative PCR (RT-qPCR) was performed on a LightCycler 96 real-time fluorescent quantitative PCR system (Bio-Rad). The SYBR Green I dye was used, and the primers for *EgHCDH* were 5′-TAG AGA TGT GGG AGC GTT GC-3′ and 5′-TCC GTA ACC GCA CTT TTT GC-3′. Expression of the actin gene was used as an internal control for normalization. Primers specific to *E. granulosus* actin were 5′-CGC ATC GGT CGT CTT GTG TT-3′ and 5′-CGG TAA TCC TGT GGC TGT CAA T-3′. The data were analyzed using the 2^−ΔΔCT^ method [13].

### Immunolocalization

To determine the tissue location of EgHCDH, fresh PSCs and adult worms were first fixed in 4% paraformaldehyde phosphate buffer overnight, followed by permeabilization with 1% Triton X-100 for 30 min and then soaking in 0.01% Triton X-100 for 1 h at 4 °C. After three washes with 0.01×  PBS, the sections were blocked in 5% (w/v) skim milk at 37 °C for 2 h, then incubated with anti-rEgHCDH mouse IgG or native mouse IgG (1:100 v/v dilutions in PBS + Tween-20 [PBST]) overnight at 4 °C, washed again and finally incubated with fluorescein isothiocyanate-conjugated goat anti-mouse IgG (H + L) (1:1000 v/v dilution in PBST) at room temperature for 2 h in the dark. After four washes with PBST, the sections were examined under a fluorescence microscope (Leica Microsystems GmbH, Wetzlar, Germany).

### Vaccination and parasite challenge

Nine beagle dogs were randomly divided into three groups. Group 1 was vaccinated with rEgHCDH mixed with Quil A adjuvant (Sigma-Aldrich); Group 2 was vaccinated with Quil A adjuvant only (Sigma-Aldrich); and Group 3 was vaccinated with PBS as a control group. One dose of vaccine comprised 200 μg of soluble rEgHCDH and 100 μg of Quil A in 825 μl of PBS. The mixture was stirred overnight at 4 °C prior to being used as vaccine. One week after the last immunization, all three groups were orally challenged with 100,000 PSCs. For safety reasons, all dogs were euthanized and necropsied at 28 days post-infection and *E. granulosus* worms were collected and counted [14].

### Indirect enzyme-linked immunosorbent assay

The enzyme-linked immunosorbent assay (ELISA) conditions were optimized by checkerboard titration of the rEgHCDH antigen and sera. The purified rEgHCDH protein (5 μg/ml) was diluted in 0.1 M carbonate buffer (pH 9.6). The ELISA plates were coated with the diluted antigen solution overnight at 4 °C, then washed with PBST and incubated with 5% skim milk for 2 h at 37 °C. The wells were then washed thoroughly and incubated with 100 μl of serum samples (1:80) in PBST at 37 °C for 1.5 h. After washing, the HRP-labeled rabbit anti-dog IgG (1:3000; Solarbio, Beijing, China) was added to the plates and incubated at 37 °C for 1.5 h, following which the wells were washed again and incubated with the substrate 3, 3′, 5, 5′-tetramethylbenzidine (Tiangen, Beijing, China) at 37 °C for 15 min. Finally, the reaction was stopped with 100 μl of 1 M H_2_SO_4_ and the optical density at 450 nm was determined.

### Detection of cytokines

The immune stimulation effect of rEgHCDH protein in dogs was evaluated using quantitative ELISA on day 28 post-challenge. Dog cytokine ELISA Quantitation kits (Jianglaibio, Shanghai, China) were used to quantify interleukin (IL)-1, IL-4, IL-5, IL-6, and interferone gamma (IFN-γ). An ELISA strip in its aluminum foil bag was left at to equilibrate at room temperature for 60 min; then the strip was removed from the bag and placed in the the standard sample well. Each well received 50 μl of dog serum at a different concentration. Next, 100 μl of HRP-labeled antibody was added to each well. The reaction wells were sealed with a sealing film and incubated at 37 °C for 60 min. The liquid was then discarded, and the plate was patted dry using absorbent paper. Each well was filled with 350 μl of PBST and left to stand for 1 min. The detergent was removed, the plate was patted dry using absorbent paper and the detergent washing step was repeated five times. Each well was then incubated with 50 μl of substrate A and 50 μl of substrate B at 37 °C for 15 min, following which 50 μl of termination solution was added to each well, and the optical density value of each well was measured at 450 nm within 15 min.

### Data analysis

All statistical analyses were performed using SPSS version 22.0 software (SPSS IBM Corp., Armonk, NY, USA). All data analyses and graphs were performed using GraphPad Prism 6.0 software package (GraphPad Software Inc., San Diego, CA, USA). Differences between groups were considered significant if the *P* value was ≤ 0.05. All experiments were repeated a minimum of three separate times.

## Results

### Molecular characteristics

The full-length cDNA sequence of *EgHCDH* consisted of 924 bp and encoded a protein of 308 amino acids (aa) with a predicted molecular mass of 34 kDa. The protein had a predicted pI of 9.03 and an instability index of 34.22. Analysis with the SignalP program revealed that the protein had a putative transmembrane domain (15–35 aa) but no signal peptide. The deduced EgHCDH sequence contains a conserved domain and belongs to the FadB family of enzymes. The structure of EgHCDH shows five α-helices around four β-folds (Additional file 1: Fig. S1). Alignment and phylogenetic analysis showed that *EgHCDH* is highly conserved with respect to its orthologs from *Echinococcus multilocularis* (Additional file 2: Fig. S2).

### Expression and purification of rEgHCDH

**Fig. 1:**
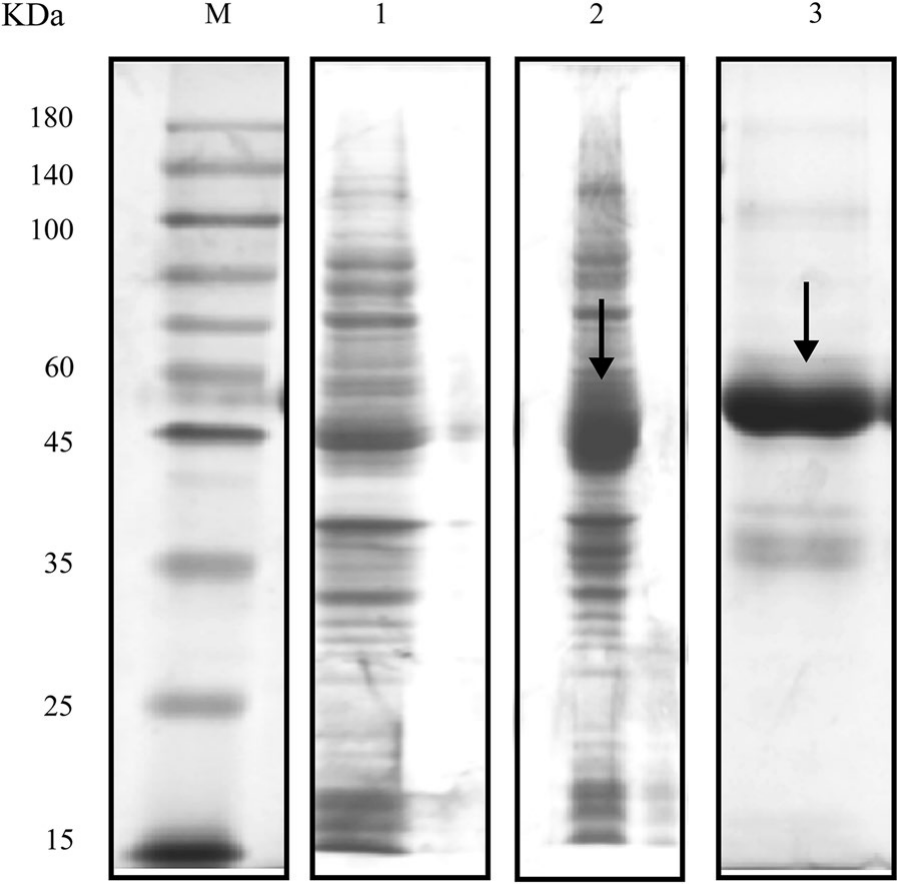
Expression and purification of recombinant (r)EgHCDH protein. Lanes: M, Protein molecular weight markers; 1, total proteins from *Escherichia coli* BL21 (DE3) transformants harboring pET32a(+)–EgHCDH; 2, total proteins from *E. coli* BL21 (DE3) transformants harboring pET32a(+)–EgHCDH induced by isopropyl-β-d-1-thiogalactopyranoside; 3, purifed rEgHCDH detected using SDS-PAGE and approximately presented the expected molecular weight of 47 kDa

The cDNA encoding the non-transmembrane region of EgHCDH was amplified and cloned into the pET32a (+) expression vector. rEgHCDH was expressed as an insoluble protein and was present in an inclusion body in *E. coli* BL21 (DE3) as a His-tag protein. Purified rEgHCDH was detected using SDS-PAGE and approximately presented the expected molecular weight of 47 kDa (Fig. 1; Additional file 3: Fig. S3).

### Western blotting

**Fig. 2:**
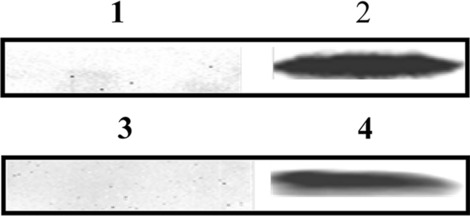
Western blot analysis. Lanes: 1, Total protein extracts of protoscoleces (PSCs) probed with preimmunized mice sera; 2, total protein extracts of PSCs probed with anti-rEgHCDH mice sera; 3, purified rEgHCDH probed with non-infected dog sera; 4, purified rEgHCDH probed with *E. granulosus*-infected dog sera

In western blotting, the native EgHCDH protein from the PSC extract could be recognized by the anti-rEgHCDH mouse IgG, and its apparent molecular mass was approximately 34 kDa, as expected. Moreover, rEgHCDH was probed by the serum from dogs infected with *E. granulosus*, and a single band of approximately  47 kDa was observed on the PVDF membrane, while no band was observed in the negative control (Fig. 2; Additional file 4: Fig. S4).

### Transcriptional profiles of *EgHCDH*

**Fig. 3:**
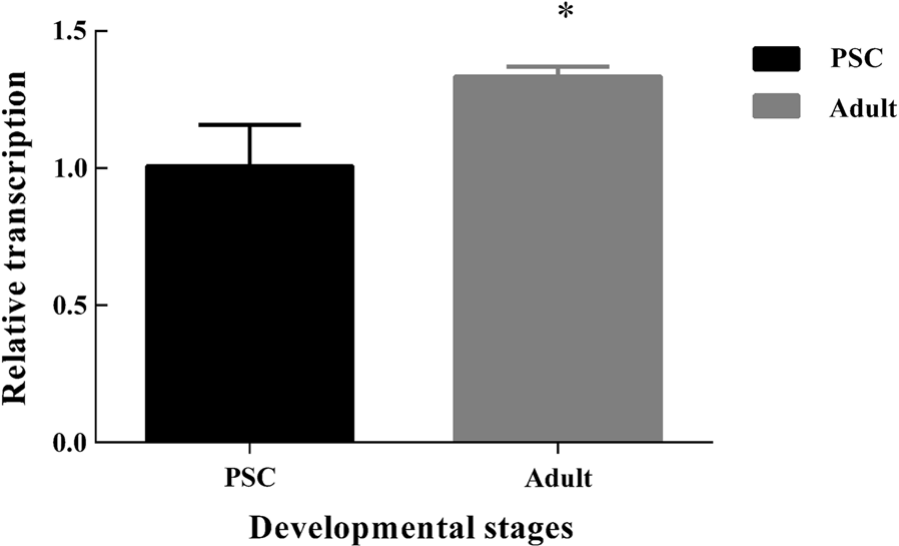
Comparison of transcript levels of *EgHCDH* gene in the PSCs and 28-day strobilated worms. Data are presented as the mean ±standard deviation (SD) of triplicate experiments. Statistically significant differences between PSCs (as the control) and 28-day strobilated worms were determined using Student’s t-test (*P < 0.05)

The *EgHCDH* gene was transcribed in both PSCs and 28-day strobilated worms, and the transcript levels of *EgHCDH* in 28-day strobilated worms were significantly higher (*P* < 0.05) than those in the PSCs (Fig. 3). (t-test: *t* = 3.67, *P* = 0.02).

### Immunolocalization of EgHCDH

**Fig. 4:**
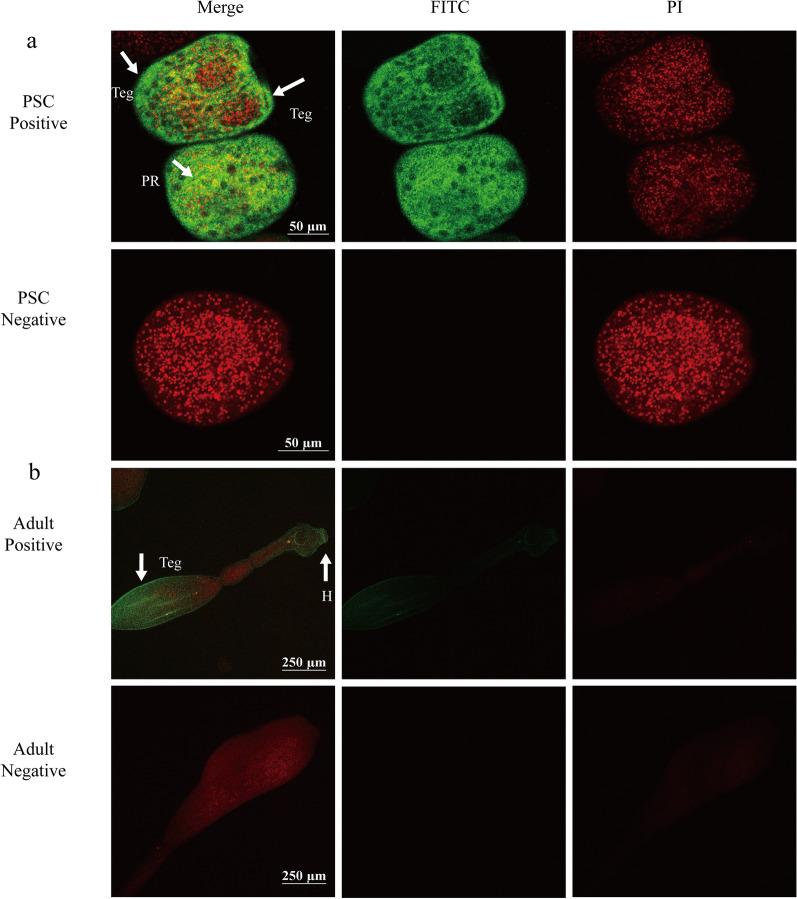
Immunofluorescent localization of EgHCDH in different stages of *E. granulosus*. The EgHCDH protein was localized in the protoscoleces and adult worms using specific anti-rEgHCDH IgG (positive) or preimmune serum (negative). Nuclei were was stained with propidium iodide (red). Abbreviations: H, Hook; PI, propidium iodide; PR, parenchymal region; Teg, tegument. Scale bars: **a** 50 µm, **b** 250 µm

The EgHCDH protein was localized in different life-cycle stages of *E. granulosus*, as detected by immunofluorescence using mouse rEgHCDH antibody. In PSCs, the fluorescence signals were mainly localized in the parenchymal region, while positive signals were also detected in the tegument tissues (Fig. 4a). In adult worms, EgHCDH was widely distributed in tegument tissue and hooks of the scolex (Fig. 4b). No fluorescence signal was detected in the negative control samples.

### IgG titers and cytokine concentrations in the sera of rEgHCDH-immunized dogs

**Fig. 5:**
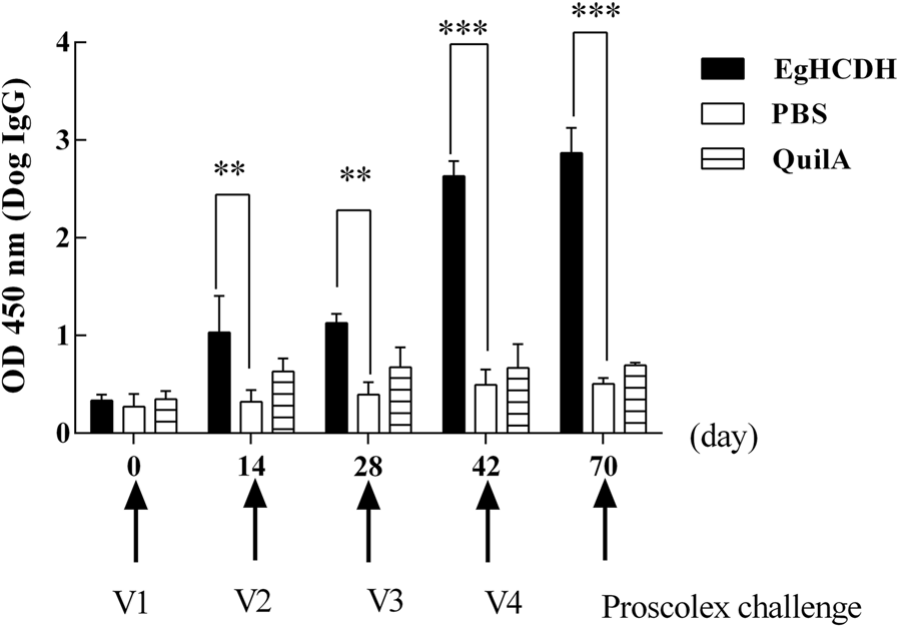
Changes in antibody IgG level in dogs. Data represent values of the immunized (rEgHCDH + Quil adjuvant) and challenged dogs compared to control groups. Plots show the changes in serum concentrations of dog IgG in the different experimental groups. Serum samples were diluted 1:100 and measured by ELISA. Asterisks indicate significant differences between groups: *P < 0.05, **P < 0.01, ***P < 0.001. Abbreviations: PBS, phosphate buffered saline

Based on indirect ELISA, the preliminary serodiagnostic potential of rEgHCDH was assessed. Sera from nine immunized dogs were measured at 14, 28 and 42 days post-vaccination. The IgG antibody responses were significantly increased in the immunized dogs after the first immunization with rEgHCDH compared with that of the control group, with the peak response detected at 42 days post-vaccination. The antibody levels gradually increased at 28 days after the protoscolex challenge and were significantly higher than those in the control group at all post-vaccination measurement time points (Fig. 5) (t-test at 14 days: *t* = 6.54, *P* = 0.0028; t-test at 28 days: *t* = 7.74, *P* = 0.0015; test at 42 days: *t* = 17.56, *P* < 0.0001; t-test after the protoscolex challenge: *t* = 17.81, *P* < 0.0001).

**Fig. 6:**
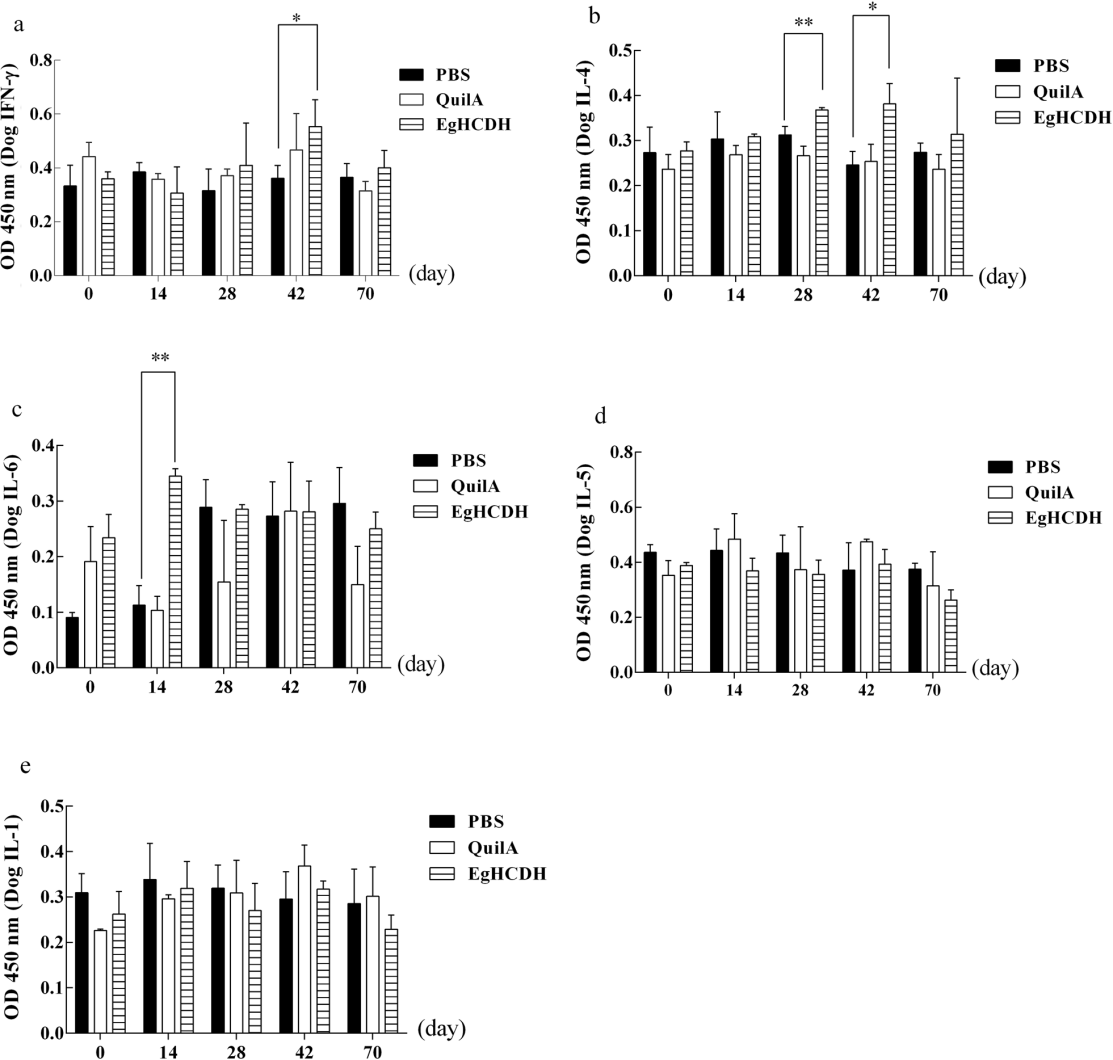
The results of ELISA for different cytokines in dogs. Data represent values of the immunized (rEgHCDH + Quil A adjuvant) and challenged dogs compared to control groups. Plots show the changes in the serum concentrations of dog IFN-γ (**a**), IL-4 (**b**), IL-6 (**c**), IL-5 (**d**) and IL-1 (**e**) in different experimental groups. Serum samples were diluted 1:100 and measured by ELISA. (* *P* < 0.05, ** *P* < 0.01, *** *P* < 0.001). Abbreviations: IL, Interleukin; IFN, interferon

The serum IFN-γ levels in the vaccinated dogs gradually increased until week 6 post-vaccination (*P* < 0.05) (t-test at 14 days: *t* = 1.32, *P* = 0.26; t-test at 28 days: *t* = 0.93, *P* = 0.41; t-test at 42 days: *t* = 3.001, *P* = 0.04), and then decreased during protoscolex challenge (*P* > 0.05) (t-test: *t* = 0.73, *P* = 0.51) (Fig. 6a). The serum IL-4 levels had significantly increased in the groups of vaccinated dogs at 4–6 weeks after first post-vaccination compared with that of the negative control (*P* < 0.01, *P* < 0.05) (t-test at 14 days: *t* = 0.13, *P* = 0.90; t-test at 28 days: *t* = 4.90, *P* = 0.008; t-test at 42 days: *t* = 4.31, *P* = 0.0126), but did not increase at 28 days after protoscolex challenge (*P* >  0.05) (t-test: *t* = 0.55, *P* = 0.61) (Fig. 6b). The serum IL-6 levels in the vaccinated dogs increased gradually from the initial vaccination until week 2 (*P* < 0.05) (t-test at 14 days: *t* = 10.57, *P* = 0.0005; t-test at 28 days: t = 0.11, *P* = 0.92; t-test at 42 days: *t* = 0.17, *P* = 0.88) and then decreased (*P* > 0.05) at 3 weeks after vaccination (Fig. 6c). There were no significant changes in the serum IL-5 and IL-1 levels in the vaccinated dogs (*P* > 0.05) (Fig. 6d, e).

### Vaccine efficacy of rEgHCDH proteins combined with Quil A

We counted the number of worms in each of the three groups. The total and average numbers of *E. granulosus* worms are shown in Table 1. Compared with the PBS (control) group, rEgHCDH-vaccinated dogs showed an 87.2% reduction in the number of *E. granulosus* worms at 28 days post-challenge. We measured the sizes of 30 randomly selected worms from each experimental group and recorded the percentage of developed (≥ four segments) versus underdeveloped (≤ three segments) worms [15]. The inhibition rate of the rEgHCDH protein on the development and maturation of worms was 67.7% compared to the development and maturation of worms in the PBS group. Thus, the rEgHCDH vaccine induced an obvious protective effect in terms of the inhibition of worm growth (Table 2).

**Table 1:** Worm burden for dogs

Experimental group	Dog no.	Number of worms	Reduction (%)^a^
Phosphate bufered saline (control) group	1	2400	
	2	3000	
	3	27,000	
Average		14,400	–
Quil A adjuvant-only group	4	13,000	
	5	3200	
	6	3800	
Average		6666	–
rEgHCDH + Quil A adjuvant griyo	7	3600	
	8	133	
	9	1800	
Average		1844	87.2
*P* value^b^			0.400

*P* value < 0.05 means that the reduction between the PBS and experimental groups was signficant according to analysis of median values

Mann–Whitney U-test: *U* = 2.000, *Z* = −1.091, *P* = 0.400

^a^Reduction % = (PBS control group average − experimental group average)/(PBS control group average) × 100

^b^The Mann–Whitney U-test was used to compare the worm burden median

**Table 2:** The development of worm segments in each experimental group

Experimental group	Dog no.	Development of the worms		Worm inhibition
		≤ 3 segments	≥ 4 segments	
rEgHCDH + Quil A adjuvant group	1	21	9	
	2	19	11	
	3	20	10	
Average		20	10	66.7%
Phosphate buffered saline (control) group	4	13	17	
	5	5	25	
	6	10	20	
Average		9	21	–
QuilA adjuvant-only group	7	3	27	
	8	9	21	
	9	18	12	
Average		10	20	–

## Discussion

Lipids are an important source of energy metabolism. The TCA cycle and β-oxidation process are the most important pathways for most organisms to obtain energy. Fatty acid β-oxidation mainly involves acetyl-CoA. In the process of β-oxidation of fatty acids, HCDH, one of the key enzymes in fatty acid metabolism, is involved in the process of removing two carbon atoms from long-chain fatty acids [9]. To further explore the role of *EgHCDH* in the development of *E. granulosus* and to evaluate its potential as a vaccine candidate antigen, this study used *EgHCDH* to lay the foundation for the successful development of a canine anti-*E. granulosus* vaccine.

The homology alignment of the EgHCDH protein sequence showed that it was in the same branch as the sequence of *E. multilocularis*, while its homology with the sequences of other species was much lower. Previous gene expression studies have shown that *EgHCDH* is consistently expressed in the larval and adult stages of *E. granulosus*, but especially in adults, suggesting that *EgHCDH* might play an important role in controlling and maintaining the specificity of the parasite life-cycle [16]. Thus, the crucial importance of TCA to *E. granulosus* and the low identity of *EgHCDH* with dog *HCDH* suggests that this molecule could be a promising target for vaccine development. Moreover, we found that *EgHCDH* was abundantly expressed in the teguments of PSCs and adult worms. It is well known that tapeworms like *E. granulosus* lack an intestinal tract and only possess microvilli or microfibrils, which are the main sites for nutrient absorption and waste excretion [17]. Thus, the distribution of EgHCDH in the tegument indicates its potential roles in nutrient absorption and metabolism. Furthermore, the location of EgHCDH in the suction cups and hooks of protocercus might be related to the energy demand while being attached to the host intestine; thus, *EgHCDH* might be involved in the interaction between the parasite and its host [18, 19]. These results also suggest that *EgHCDH* plays a key role in the growth and physiological activities of *E. granulosus* [20]. The western blotting results showed that the serum from mice injected with rEgHCDH could recognize EgHCDH among the natural proteins of PSCs and that the positive serum from dogs infected with *E. granulosus* could also recognize rEgHCDH; these results indicate that rEgHCDH had good immunoreactivity. To our knowledge, if a protein can be recognized by naturally infected host serum, it indicates that the protein either contains a signal peptide or has exosome secretion [17, 21]. However, the results showed that the predicted amino acid sequence of EgHCDH did not contain a signal peptide, which indicated that EgHCDH might be a component of the excretory/secretory (ES) products of *E. granulosus*.

In echinococcosis, some studies have shown that Th1-type and Th2-type cytokines coexist [22, 23]. Th2 cytokines can induce a protective humoral immune response and play a key role in the parasite escape from immune surveillance [24]. IL-4 is a key cytokine. This is an important step in the development of a prophylactic *E. granulosus* vaccine [25]. In our study, compared with the PBS control group, rEgHCDH showed a high effectiveness in dogs, and the IL-4 level in the serum of the immunized dogs increased significantly until day 42 post-inoculation (*P* < 0.05). This suggests that rEgHCDH mixed with QuilA could be effective for the induction of Th2 immune responses, although there was a downward trend 28 days after challenge infection. Studies on the *E. granulosus-*infected dogs have shown a defined Th2-type cytokine which, possibly, may suppress the growth and development of PSCs to the adult worms, which is remarkable [26, 27]. The Th2 immune response might also play an important role in the anti-parasite response [28]. In comparison, IFN-γ is one of the key Th1-type cytokines that can enhance the expression of MHC-II molecules and antigen presentation ability, thus inhibiting the growth of protoscolex of *E. granulosus* [29]*.* These results support the view that rEgHCDH plays an important role in anti-*E. granulosus* infection.

The Quil A adjuvant has been proven to be a key component of current vaccines. Adding adjuvants to vaccine candidates could enhance the efficacy of the antigen, reduce the required dosage of the vaccine, facilitate a more rapid induction of the host immune response, enable T helper cells bind more effectively to optimize the quality and persistence of the antibody response or induce effector CD4+ or CD8+ T cells to kill pathogens in cells [30]. Therefore, this study used rEgHCDH mixed with the Quil A adjuvant to induce a more effective immune response. After the third immunization, 100,000 PSCs were administered orally in the PBS group as the control. The IgG titers in each immunization group were monitored. We observed that the antibody levels of the vaccine group increased gradually with prolonged immunization time, indicating that specific antibodies were produced by stimulation with rEgHCDH. In the present study, all dogs were sacrificed at 28 days post-challenge, before the appearance of eggs, for safety reasons. Compared with the PBS control group, the worm reduction rate was 87.19% in the rEgHCDH group, which is higher than that reported for the* Salmonella* vaccine EgA31-EgTrp (70%–80%) [15], but slightly lower than that of the recombinant vaccine EgM123 (89.2%) [30]. Furthermore, worm development to the mature stage was significantly decreased in the rEgHCDH group, with an inhibition rate of 67.7%, which is lower than that reported for the recombinant vaccine EgM123 [14]. These results indicate that rEgHCDH could inhibit the growth and development of tapeworms in canine intestines, which supports rEgHCDH as a potential vaccine candidate against *E. granulosus* infection.

## Conclusions

This study is the first to evaluate the immunoprotective effect of rEgHCDH protein against *E. granulosus* infection in canines. The results showed that rEgHCDH could induce specific antibodies in dogs, effectively reduce the worm burden and inhibit the development of tapeworms, indicating that rEgHCDH is a potential candidate vaccine for canines against *E. granulosus* infection.

## Supplementary Information

**Additional file 1: Fig. S1.** Prediction of the three-dimensional structure of the EgHCDH protein.

**Additional file 2: Fig. S2.** Phylogenetic tree of *EgHCDH*. The evolutionary tree was constructed using the neighbor-joining method in MEGA software.

**Additional file 3: Fig. S3.** Expression and purification of rEgHCDH protein.** a** Lanes: M, Protein marker; 1, recombinant plasmid (pET-32a empty vector) was transferred to BL21 induced by isopropyl-1-thio-β-d-galactopyranoside (IPTG) for 6 h; 2–6, rRecombinant plasmids (pET32a–EgHCDH) were transferred to BL21 induced by IPTG for 0, 2, 4, 6 and 8 h.** b** Lanes: M, Protein marker; 1–2, purified protein.

**Additional file 4: Fig. S4.** Western blot analysis. Lanes: 1, Total protein extracts of PSCs probed with preimmunized mice sera; 2, total protein extracts of PSCs probed with anti-rEgHCDH mice sera; 3, purified rEgHCDH probed with non-infected dog sera; 4, purified rEgHCDH probed with *E. granulosus*-infected dog sera.

## Data Availability

The full-length DNA sequence of *EgHCDH* was deposited in the GenBank database (https://www.ncbi.nlm.nih.gov/) under the accession number XP_024352997.1. Data supporting the conclusions of this study are included in the article. The data used and/or analyzed during the current study are available from the corresponding author upon reasonable request.

## Abbreviations

CE: Cystic echinococcosis
*EgHCDH*: *Echinococcus granulosus* 3-hydroxyacyl-CoA dehydrogenase
ELISA: Enzyme-linked immunosorbent assay
SDS-PAGE: Sodium dodecyl sulfate–polyacrylamide gelelectrophoresis
