# Supplementary figures and images for: Molecular characterization and immune protection of the 3-hydroxyacyl-CoA dehydrogenase gene in Echinococcus granulosus
Source: Parasit Vectors. 2021 Sep 23;14:489. doi: 10.1186/s13071-021-05001-z (PMC8460197; doi:10.1186/s13071-021-05001-z)

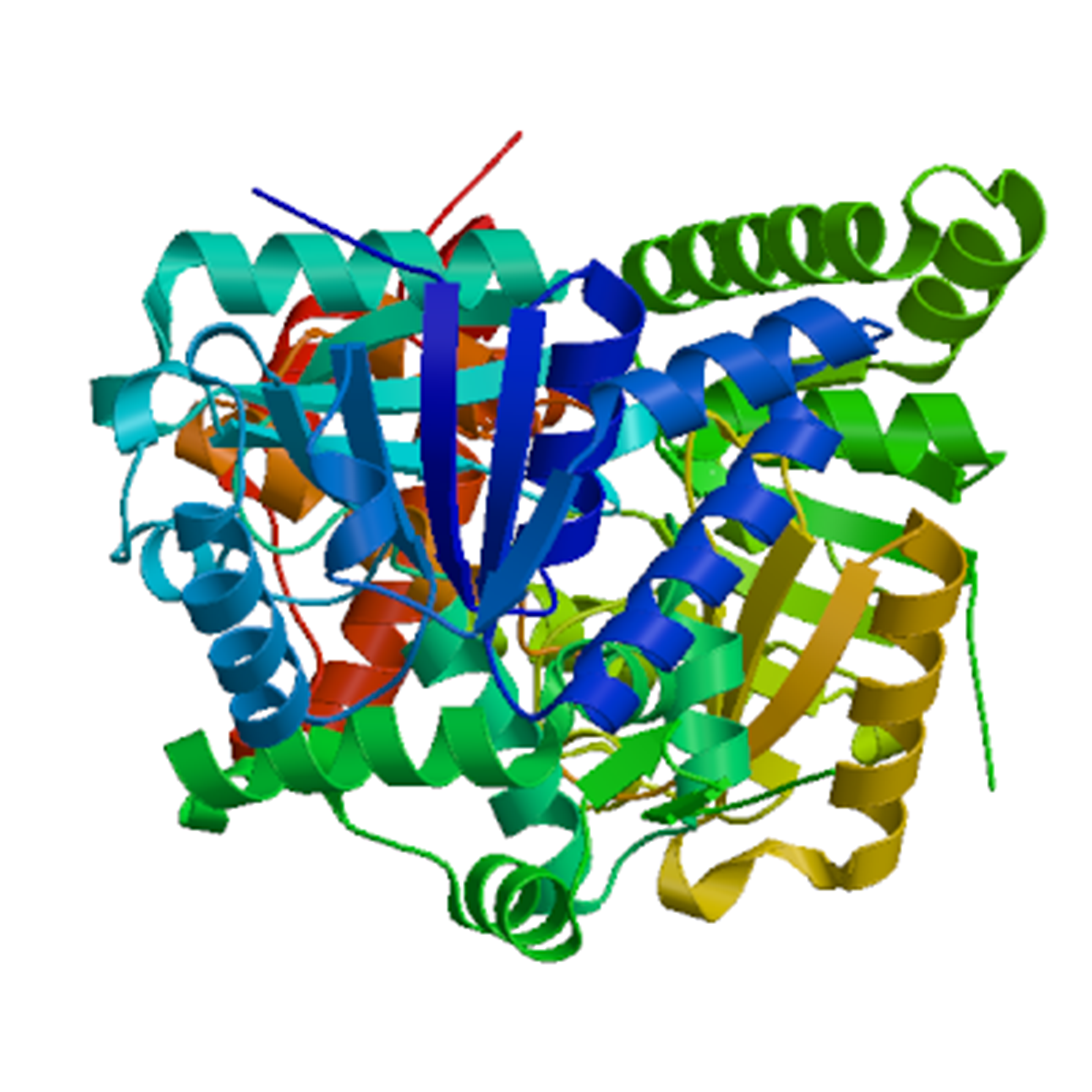

Supplement: Supplementary file 1 — Additional file 1: Fig. S1. Prediction of the three-dimensional structure of the EgHCDH protein. [file 13071_2021_5001_MOESM1_ESM.tif]

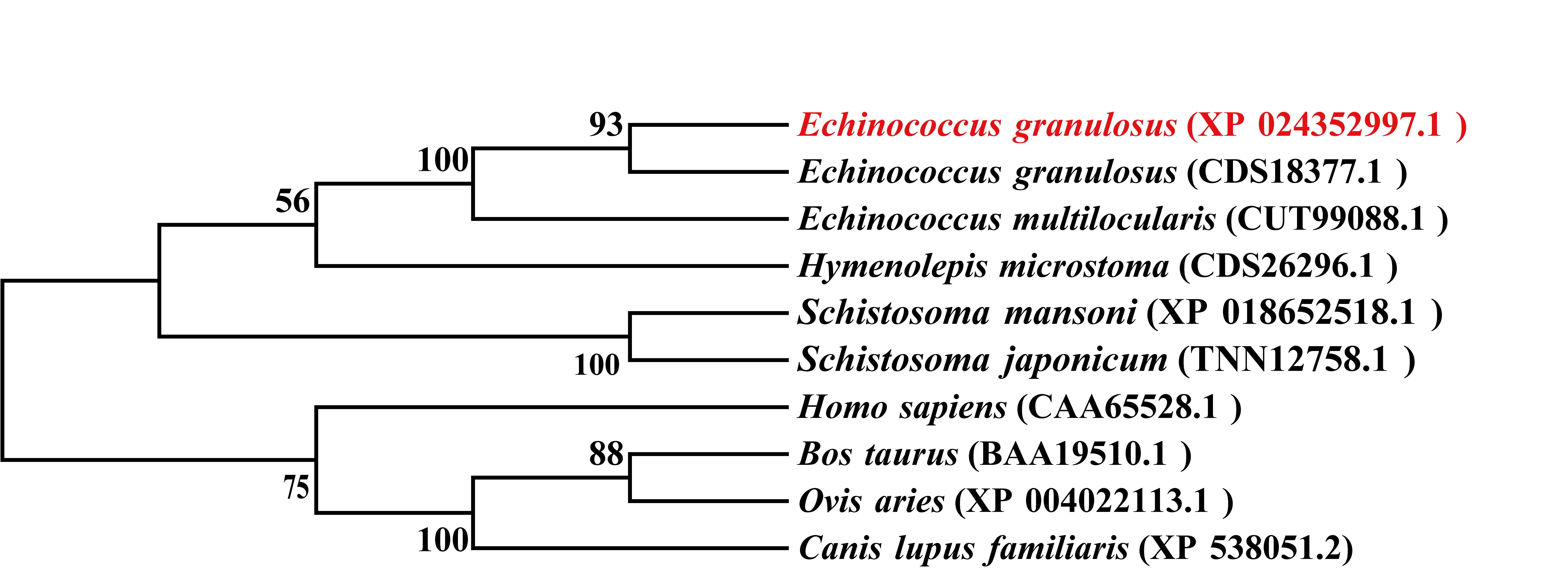

Supplement: Supplementary file 2 — Additional file 2: Fig. S2. Phylogenetic tree of EgHCDH. The evolutionary tree was constructed using the neighbor-joining method in MEGA software. [file 13071_2021_5001_MOESM2_ESM.tif]

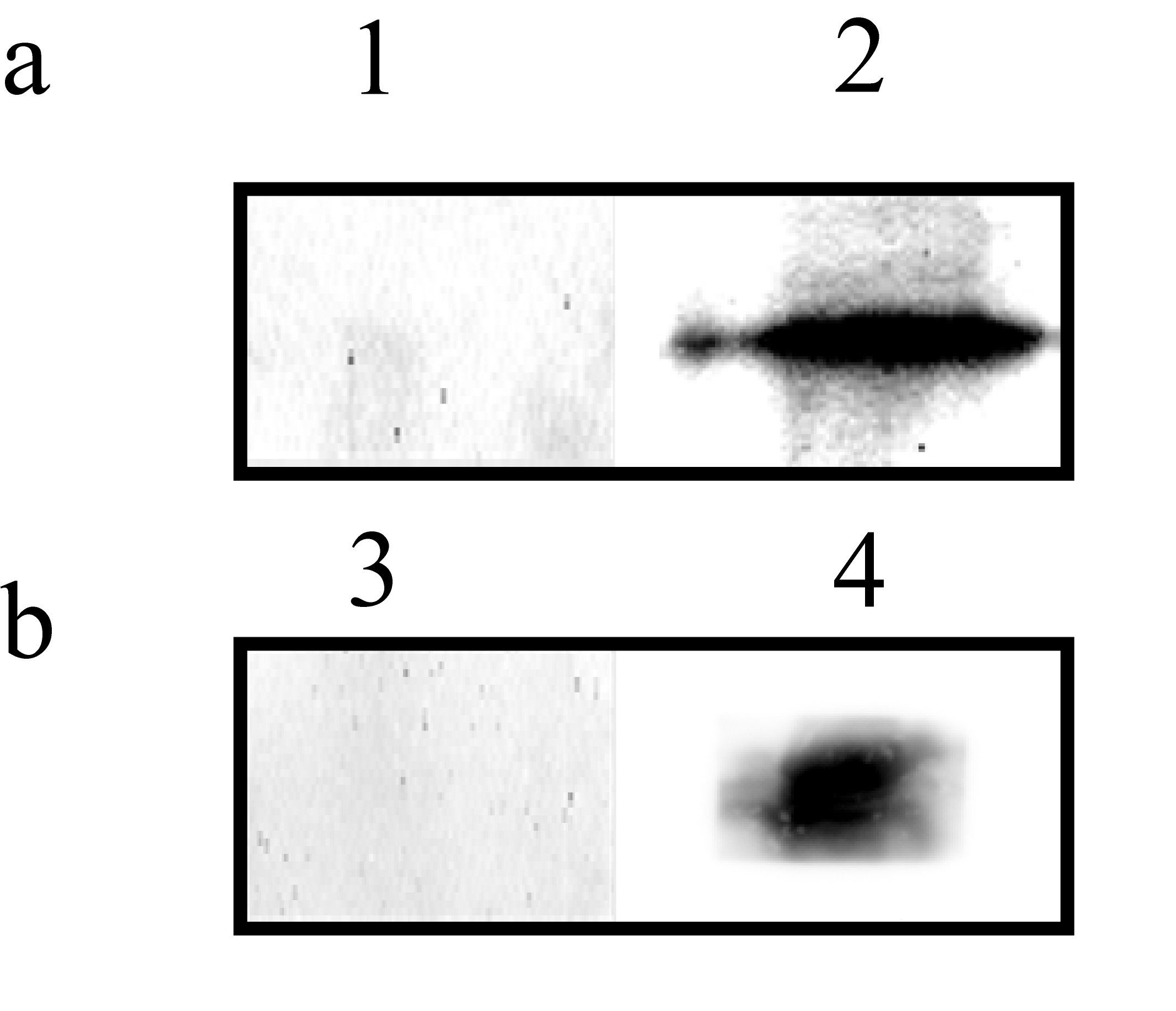

Supplement: Supplementary file 4 — Additional file 4: Fig. S4. Western blot analysis. Lanes: 1, Total protein extracts of PSCs probed with preimmunized mice sera; 2, total protein extracts of PSCs probed with anti-rEgHCDH mice sera; 3, purified rEgHCDH probed with non-infected dog sera; 4, purified rEgHCDH probed with E. granulosus-infected dog sera. [file 13071_2021_5001_MOESM4_ESM.tif]
